# Donor acquired visceral leishmaniasis following liver transplantation

**DOI:** 10.1136/flgastro-2020-101659

**Published:** 2021-01-04

**Authors:** Amritpal Dhaliwal, Abhishek Chauhan, Dinesh Aggarwal, Pretin Davda, Miruna David, Rasoul Amel-Kashipaz, Rachel Brown, Martin Dedicoat, Fiona Clark, Tahir Shah, Ahmed Mohamed Elsharkawy, Ines Ushiro-Lumb, Peter Chiodini, Omar El-Sherif, Matthew Armstrong, James W Ferguson

**Affiliations:** 1 Liver Unit, Queen Elizabeth Hospital Birmingham, Birmingham, UK; 2 National Institute for Health Research (NIHR) Biomedical Research Centre Birmingham, University of Birmingham, Birmingham, UK; 3 Hospital for Tropical Disease, University College London, London, UK; 4 Department of Microbiology, University Hospitals Birmingham NHS Foundation Trust, Birmingham, UK; 5 Department of Histopathology, University Hospitals Birmingham NHS Foundation Trust, Birmingham, UK; 6 Department of Haematology, University Hospitals Birmingham NHS Foundation Trust, Birmingham, UK; 7 Department of Haematology, NHS Blood and Transplant, Watford, UK; 8 Liver Medicine, Queen Elizabeth Hospital Birmingham, Birmingham, UK; 9 National Institute for Health Research (NIHR), Biomedical Research Centre, University of Birmingham, Birmingham, UK

**Keywords:** liver transplantation

## Abstract

Patients who undergo solid organ transplantation are at risk of opportunistic infection associated with immunosuppression. We report a case of confirmed donor derived visceral leishmaniasis (VL), in a patient following liver transplantation causing fever and pancytopenia. The diagnosis was confirmed by bone marrow biopsy, with confirmed positive donor serology, with no other route of transmission. To our knowledge, this is the first case report in the United Kingdom and Europe, of confirmed organ donor transmission of VL. This case report highlights an important consideration of donor derived infections, in the context of solid organ transplantation.

## Introduction

Visceral leishmaniasis (VL) is parasitic infection common occurring in Asian, African and Central and South Americas. It often presents with fever, splenomegaly and pancytopenia. This clinical presentation is regularly observed in patients post liver transplantation. Common aetiologies for this include, graft rejection, immunosuppressive agents and opportunistic infections such as cytomegalovirus (CMV).

This case describes donor derived leishmaniasis as a rare cause of fever and pancytopenia following liver transplantation in a patient from the UK. It highlights the importance to consider other less frequent causes of infection, including donor derived infections in those following solid organ transplantation.

## Case history

A 57-year-old man underwent orthoptic liver transplantation in March 2019 for alcoholic-related liver disease and portal hypertension, following 2 years of abstinence. The patient was born in Wales and his only travel history outside the UK was a transient visit to Calais, 15 year prior to his transplantation. He was a carpenter and did not work near the ports or places of travel in Wales.

He required a 2-day intensive care admission following transplantation from a brainstem death donor. Immunosuppression was commenced with prednisolone, tacrolimus and mycophenolate (MMF) in conjunction with cotrimoxazole prophylaxis. Both the donor and recipient were CMV IgG negative, therefore, CMV prophylaxis was not required as per hospital policy.

He developed an episode of biopsy-proven moderate acute cellular rejection requiring treatment with 3 days of pulsed steroids 1-month postliver transplant. His graft function gradually improved, with normalisation of alanine aminotransferase (ALT) coinciding with the development of pancytopenia 4 months post-transplantation. His MMF was reduced from 1 g two times a day to 500 mg two times a day in response to this.

Despite this, the patient developed a worsening neutropenia and persistent pancytopenia, with the additional systemic symptoms of fever and rigours. He then was admitted to his local hospital with neutropenic sepsis and an acute kidney injury. He was transferred from his local hospital to our liver transplant centre for further investigation.

He underwent a further series of investigations as summarised in table 1, during his admission. He was treated empirically with meropenem, vancomycin and fluconazole.

**Table 1:** Summary of pertinent investigations performed throughout this case

Haematology	Microbiology & Virology	Imaging
Folate deficiency Iron deficiency	HSV PCR negative HHV6 negative Varicella zoster negative HSV type 1 and type 2 negative Enterovirus RNA negative Adenovirus DNA negative EBV PCR <150 IU/mL CMV PCR <200 copies/ml Stool MC&S negative Parvovirus PCR negative Cryptococcal antigen negative Toxoplasma IgG negative Legionella antigen-negative Mycobacterial blood cultures-negative Galactomannan-positive test 0.26 Beta glucan positive 119 pg/mL Serum Leishmania PCR negative Leishmaniasis serology positive titre ≥1:102 400	MRCP: 22 cm splenomegaly, no intrahepatic dilatation, upper abdominal varices CT thorax, abdomen and pelvis: right sided pleural effusion, splenomegaly Echocardiogram: no evidence of vegetations CT PET: massive splenomegaly with increased uptake
	**Pleural fluid and broncho alveolar lavage** Broncho alveolar lavage negative aspergillus PCR Pleural aspirate: ph 7.4, exudate, MC&S negative Acid fast bacilli not seen Mycobacterium negative	
Bone marrow aspirate: Small atypical B cells, amastigotes present	**Bone marrow** Leishmania PCR positive 16S DNA-no DNA detected Pneumocystis jirovecii PCR-negative Toxoplasma PCR-negative Bacterial MC&S-negative Panfungal PCR-negative	

CMV, cytomegalovirus; CT, computerised tomography; CT PET, computerised tomographic- positron emission tomography; DNA, deoxyribonucleic acid; EBV, Epstein-Barr virus; HHV 6, human herpes virus 6; HSV, herpes simplex virus; IgG, immunoglobulin G; MC&S, microscopy, culture & sensitivity; MRCP, magnetic resonance cholangiopancreatography; PCR, polymerase chain reaction; RNA, ribonucleic acid.

**Figure 1:**
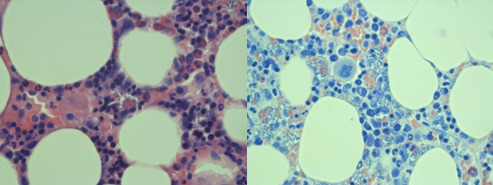
Bone marrow H&E stain versus bone marrow long giemsa stain ×60.

A bone marrow biopsy was performed in view his pancytopenia and it showed amastigotes (see figure 1). The *Leishmania* PCR test detected DNA from the *Leishmania donovani* complex. Furthermore, the patient’s *Leishmania* serology was strongly positive at a titre equal to or greater than 1 in 102 400 (a titre of 1 in 1600 or above is considered positive). His peripheral blood PCR for *Leishmania* was negative.

He was given liposomal amphotericin (AmBisome) at a dosage of 4 mg/Kg for 21 days, with further doses at 24, 31 and 38 days to treat his leishmaniasis. Treatment of VL in immunocompromised patients is with Liposomal amphotericin B4 mg/kg daily on days 1 to 5, 10, 17, 21, 31 and 38, however, he received the longer regimen to maintain empirical antifungal cover. He underwent a repeat bone marrow biopsy following 6 days of treatment with AmBisome. This showed ongoing leishmanial infection, evidence of secondary haemophagocytosis and reduced myeloid activity.

His fevers had settled and his pancytopenia slowly improved. He was later repatriated to his local hospital when his pancytopenia resolved. He was maintained on tacrolimus monotherapy immunosuppression for his liver transplant.

**Figure 2:**
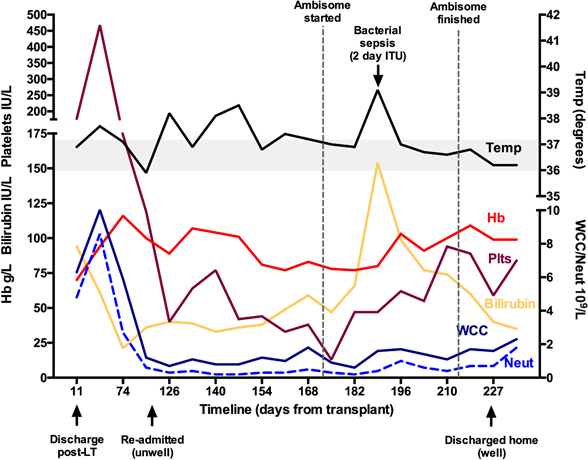
Trajectory of the blood count and corresponding temperature over the admission. Hb, haemoglobin; ITU, intensive care unit; LT, liver transplantation; WCC, white cell count.

Figure 2 shows the trajectory of his blood count and corresponding temperature over his admission period.

He had not travelled abroad post operatively, therefore, reactivated or newly acquired leishmanial infection was extremely unlikely, however this was considered.

The donor samples were investigated by National Health Service (NHS) Blood and Transplant to explore all routes of transmission. Leishmanial serology on serum from the liver donor was positive. Additionally, it transpired that the donor had travelled to India in the year preceding his death, and therefore the most likely transmission in this case is via the donor.

## Visceral Leishmaniasis

**Figure 3:**
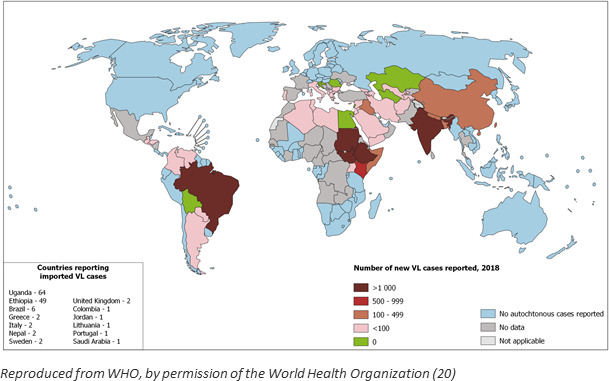
Status of endemicity of visceral leishmaniasis worldwide in 2018.

VL is an obligate intracellular protozoal parasitic infection transmitted by sand flies (*Phlebotomus spp in the Old World and Lutzomyia spp in the New World).* 1 Leishmaniasis manifests in three forms, cutaneous, mucosal and visceral, determined by infecting species and host response. *VL is caused by the Leishmania donovani* complex: *L. donovani* in Asia and Africa; *L. donovani infantum* in the Mediterranean Basin, Africa, the Middle East, central Asia and China; and *L. donovani chagasi* (a synonym of *L. donovani infantum*) in Central and South America.^2–4^ VL is fatal in 80%–90% of those left untreated. Cases diagnosed in the UK are usually de novo infections acquired during travel. Figure 3 shows those areas currently regarded as endemic for VL.^5^

In an immunocompromised individual, any conditions supressing host T cell responses increase the risk of reactivation. Leishmaniasis following liver transplantation can occur via the following routes; (1) Donor infection via the graft which is extremely rare with no confirmed case reports in non-endemic countries, (2) Via blood transfusion which has been reported in endemic and non-endemic countries,^6 7^ (3) Reactivation of previous infection in recipient,^8^ (4) De novo vectorborne infection in the individual, usually from travel to an endemic country.^9 10^

The clinical presentation is characterised by persistent fluctuating fever, and splenomegaly. Other features include pancytopenia, hepatomegaly, weight loss, cough, diarrhoea and hypergammaglobulinaemia.^4 11^ Malnutrition and muscle wasting is associated with a high parasite burden.^12^ The onset of VL can be acute or gradual, with an incubation period of 2–8 months. If left untreated, VL is usually fatal within 2 years. Emaciation sets in and death is often caused by one or more secondary infections.^4 11^ Immunocompromise due to HIV, autoimmune disease, treatment with biological agents or other immunosuppression, and diabetes predisposes patients to more serious infections of VL.

VL is diagnosed through identifying amastigotes in tissue (figure 1) or flagellated promastigotes in culture. Splenic aspirates are the gold standard with a sensitivity of >95%. In experienced hands, splenic aspiration is safe provided the patient has a normal prothrombin time and a platelet count greater than 40×10^9^/L. Bone marrow aspiration is more commonly used and detects 85% of cases.

PCR should be performed in addition to microscopy on all samples. PCR on peripheral blood is useful for follow-up of VL in immunocompromised patients, but the yield from peripheral blood samples is too low for use in primary diagnosis in those with intact immunity.^13^

The drug of choice for VL is liposomal amphotericin B, with efficacy rates of >90%.^4 14 15^ Most patients usually recover. Mortality is 80%–90% if left untreated, <5% if treated.^2 14^ Antimonials, or paromomycin, or miltefosine are alternatives if liposomal amphotericin B is contraindicated or unavailable.

## Discussion

This case highlights donor-derived VL as a potential cause of pancytopenia after liver transplantation. We have performed over 5000 liver transplants in our centre and this is the first case of VL transmission via a donor. It is the first reported case in the literature of donor contracted Leishmaniasis in the setting of liver transplantation, as evidenced by positive donor serology for *Leishmania* on retrospective evaluation and a lifetime travel history incompatible with de novo vector-transmitted or reactivated infection in the recipient. A recent case report from Brazil highlights a case of VL post liver transplantation via reinfection, from a patient in an area where VL is endemic.^16^ Our case raises several questions regarding the medical and travel history of organ donors, storage of their tissue and blood samples in case retrospective testing is required, notification measures and whether screening measures may need to be reconsidered.

Due to the acute nature of organ donation, obtaining a full history, including travel can understandably prove difficult. Often this is carried out retrospectively, as and when required, from collateral sources. Storage of tissue and blood samples from organ donors is currently performed; they are stored for up to several years, but given the anonymity of donation, this is done by NHS Blood and Transplant with restricted access. In this case, we were able to obtain a donor travel history and serum samples which were tested. It is important that such provisions, for storage to occur.

Transfusion-related transmission has been described in earlier reports.^17^ This is extremely unlikely in our patient. Recent changes to blood donation regulations in 2017 have ensured a stringent safety check on donating blood, including a donor health check, private health screening, in addition to screening for syphilis, hepatitis B virus, hepatitis C virus, hepatitis E virus, HIV and in first time donors Human T-lymphotropic virus. Additional tests such as malaria serology may be performed if pertinent. Leucodepletion is effective in removing *Leishmania* from peripheral blood^18^ and is used routinely in UK blood banks. Therefore, transmission through this vector is extremely unlikely.

In countries where Leishmaniasis is endemic, routine donor screening pre-liver transplantation is not recommended given the limited data on donor-derived infection^19–21^; this may require reconsideration with the increase in organ transplantation, and worldwide travel and migration.^22^

While our patient had classical features of VL, with persistent fever and pancytopenia, persistent splenomegaly is common following liver transplantation in a patient with portal hypertension and therefore can be overlooked in this setting. It highlights the need for a wider differential diagnosis in post transplantation patients.^23^ Additionally, confirmation of VL requires bone marrow for microscopy, culture and PCR, which provide far greater sensitivity than peripheral blood. Therefore, when confronted with a possible clinical suspicion of VL, in all cases, especially in an immunocompromised patient, negative serology alone should not be relied on to exclude it. In support of this, other publications have reported positive bone marrow or lymph node aspirates in the absence of positive serology.^10^
